# Does housing system affect horse welfare? The AWIN welfare assessment protocol applied to horses kept in an outdoor group-housing system: The ‘parcours’

**DOI:** 10.1017/awf.2023.9

**Published:** 2023-02-27

**Authors:** Francesca Dai, Emanuela Dalla Costa, Michela Minero, Christine Briant

**Affiliations:** 1 Università degli Studi di Milano, Dipartimento di Medicina Veterinaria, e Scienze Animali, via dell’Università 6, 26900 Lodi, Italy; 2 PRC, INRAE, CNRS, IFCE, University of Tours, Nouzilly, France

**Keywords:** animal welfare, AWIN, group-housing, horse, management, ‘parcours’

## Abstract

Outdoor group housing is generally reported as being beneficial to the welfare of horses compared to single boxes, being considered to show greater similarities with the living conditions of feral horses, allowing full expression of behaviours such as grazing, social interactions and free movement. However, concerns persist regarding the ability to maintain a good nutritional state and the possibility of acquiring injury. No data reporting a comprehensive assessment of welfare for horses in outdoor group-housing systems are currently available. The present study aimed at applying a scientifically valid welfare assessment protocol to group-housed outdoor horses in ‘parcours’, a particular management system used in the south of France. ‘Parcours’ are semi-natural areas, grazed by domestic herbivores located in lowland, mountain, or marsh. One hundred and seventy-one horses older than a year pertaining to six farms and kept on ‘parcours’ were evaluated by a trained veterinarian using a modified version of the second level Animal Welfare Indicators (AWIN) welfare assessment protocol for horses. No major welfare issues were detected. Horses in ‘parcours’ displayed few abnormal behaviours, they could move freely for most of the day and interact with conspecifics, maintaining a healthy state of nutrition and a good relationship with humans. The main welfare concerns were related to the presence of superficial integument alterations such as alopecia, difficulty in reaching quality controlled water sources and a lack of shelter. As the number of facilities involved in this study is relatively limited, further harmonised data collection should aim to enlarge the sample size and allow comparison with different outdoor group-housing conditions.

## Introduction

The adaptability of horses sees them bred for many different types of activity (e.g. breeding, non-competitive recreational riding, leisure and sport, education) and kept in a variety of different housing and management conditions that could potentially impact upon their welfare (McGreevy 2004; Visser *et al*. 2014; Dalla Costa *et al*. 2017b).

The most common housing system in Western countries is single boxes (Thorne *et al*. 2005; Dalla Costa *et al*. 2014b) with the literature stating that the proportion of sport horses stabled in single boxes ranges from 32 to 90% in different nations (Hotchkiss *et al*. 2007; Leme *et al*. 2014; Visser *et al*. 2014; Hockenhull & Creighton 2015; Larsson & Müller 2018). Welfare scientists consider single-box housing to present unfavourable aspects for horse welfare (Ruet *et al*. 2019) since confinement prevents horses from engaging in highly motivated behaviours such as movement (Chaplin & Gretgrix 2010), social relationships (Søndergaard & Ladewig 2004) and natural feeding behaviour. As herbivores, grazing occupies up to 16 h of the feral horse’s day (Souris *et al*. 2007; Hampson *et al*. 2010a, b) while horses kept in single boxes traditionally have restricted access to high-fibre forage and their diet includes energy-dense cereal grains for ingestion more quickly (Jansson & Harris 2013). This daily ration can be consumed in less than 3 h (McGreevy *et al*. 1995), leaving horses without food for a large portion of their day, which could contribute to the development of stereotypies and gastric ulcers (Hoffman *et al*. 2009); moreover, decreased exposure to pasture is reported to be a risk factor for the onset of colic (Hudson *et al*. 2001). To better meet horses’ needs, in recent years, a shift to a diet higher in forages has been observed (Jansson & Harris 2013). Modern horse-feeding habits vary from country-to-country and most owners report feeding their horses a forage-based diet, supplemented with concentrated feeds (Hoffman *et al*. 2009; Auriane Hurtes 2015; Murray *et al*. 2015; Kaya-Karasu *et al*. 2018; Larsson & Müller 2018), however *ad libitum* access to roughage still occurs infrequently (Kaya-Karasu *et al*. 2018; Larsson & Müller 2018). Horses are a social species (Mills & Nankervis 1999), but traditional single boxes prevent them interacting freely with conspecifics, making it impossible to form cohesive social bonds. To overcome this, alternative box housing designs have been used. An alternative design sees stalls where the partition between two boxes is made up of a solid lower segment and an upper part consisting of vertical metal bars; this allows both visual and olfactory contact while limiting tactile contact (Gmel *et al*. 2022). So-called ‘social boxes’ are neighbouring stalls separated by ‘social bars’ (full height vertical bars spaced at 30 cm) making up half the partition and enabling visual, auditory, olfactory, and physical contact, while a solid partition also allows horses to withdraw (Gehlen *et al*. 2021; Gmel *et al*. 2022). Unfortunately, little experimental work has been done addressing how well these alternative housing systems function (Hartmann *et al*. 2012). Dalla Costa and colleagues (2017b) highlighted that only 9.8% of horses in Europe are able to nibble and partly groom conspecifics and 22.3% have zero opportunity for social contact; visual or olfactory. Frustration, induced by the fundamental restrictions imposed by such housing causes a high proportion of horses to develop some kind of undesired behaviours (McGreevy *et al*. 1995; Cooper & Albentosa 2005): the reported prevalence of stereotypies in horses kept in boxes ranges from 14.4 to 32.5% (McGreevy *et al*. 1995; Sarrafchi & Blokhuis 2013; Muñoz *et al*. 2014; Ruet *et al*. 2019).

Outdoor group housing (e.g. paddock or pasture) may be considered to have greater similarities with the living conditions of feral horses. Scientific research supports that housing animals in more natural conditions (e.g. group housing) can improve their welfare (for a review, see Fraser 2009). In fact, more natural housing conditions allow animals to perform species-specific behaviours freely, but, on the other hand, could threaten their welfare by enhancing the possibility of developing injuries and illnesses (Fraser 2008, 2009) and the reducing human-animal bond. As for horses, outdoor group housing is generally considered less practical for the caretaker, and potentially dangerous for horse health (McGreevy 2004): by stabling their horses, owners consider themselves better able to manage nutrition, parasitic control, coat care, protection from atmospheric agents, while reducing the risks of aggressive interactions with other horses and the need for the horse to work for food (McGreevy 2004). However, to date, no scientific data reporting a global assessment of welfare for horses in outdoor group-housing are available. In the south of France, a particular type of outdoor group-housing system entitled ‘parcours’ is traditionally adopted. ‘Parcours’ are semi-natural areas, grazed by domestic herbivores; consisting of spontaneous lawn, moor and wood proliferation located in areas of lowland, mountain, or marsh. Breeders explain that horses can eat grass, but also leaves or tree branches. In fact, horses were found to spend as much as 18% of their feeding time consuming such resources (Etienne *et al.* personal communication 2020). Horses, therefore, can contribute to the maintenance of these uncultivated areas, perhaps helping the prevention of vegetation fires. In fact, in the south of France, sheep are commonly used to maintain pastoral areas, by consuming plants liable to catch fire and by opening paths, which act as firebreaks. A recent study discussed how animals, including herbivores, can affect fire behaviour by modifying the amount, structure, or condition of fuel, as they eat those parts of the trees and bushes most likely to catch fire (Foster *et al*. 2020). Thus, ‘parcours’ are considered environmentally sustainable, but an evaluation of the welfare of the horses kept in this management condition is necessary.

Horse welfare assessment could be based on the collection of animal-, resource- and/or management-based indicators. Animal-based indicators relate directly to the animal itself rather than to the environment in which the horse is kept (EFSA Panel on Animal Health and Welfare 2012), therefore these indicators can be collected in different housing conditions and used to infer how the animal is affected by external factors such as housing system. The AWIN welfare assessment protocol for horses, based on the Welfare Quality® principles and criteria, includes 25 animal-, resource- and management-based indicators (Dalla Costa *et al*. 2016). The protocol has been applied by Dalla Costa *et al*. (2017b) to collect welfare data in 355 single-stabled horses in Italy and Germany. Some adaptation to the AWIN protocol was suggested by the authors for assessing the welfare of horses kept in groups, however, to the authors’ knowledge, no specific data collection using the AWIN protocol on outdoor group-housed horses was published.

The aim of the present work was to collect data on the welfare of horses housed in a specific outdoor group-housing system known as ‘parcours’ via application of a complete and comprehensive welfare assessment method (the AWIN welfare assessment protocol for horses).

## Materials and methods

### ‘Parcours’ description

**Figure 1.**
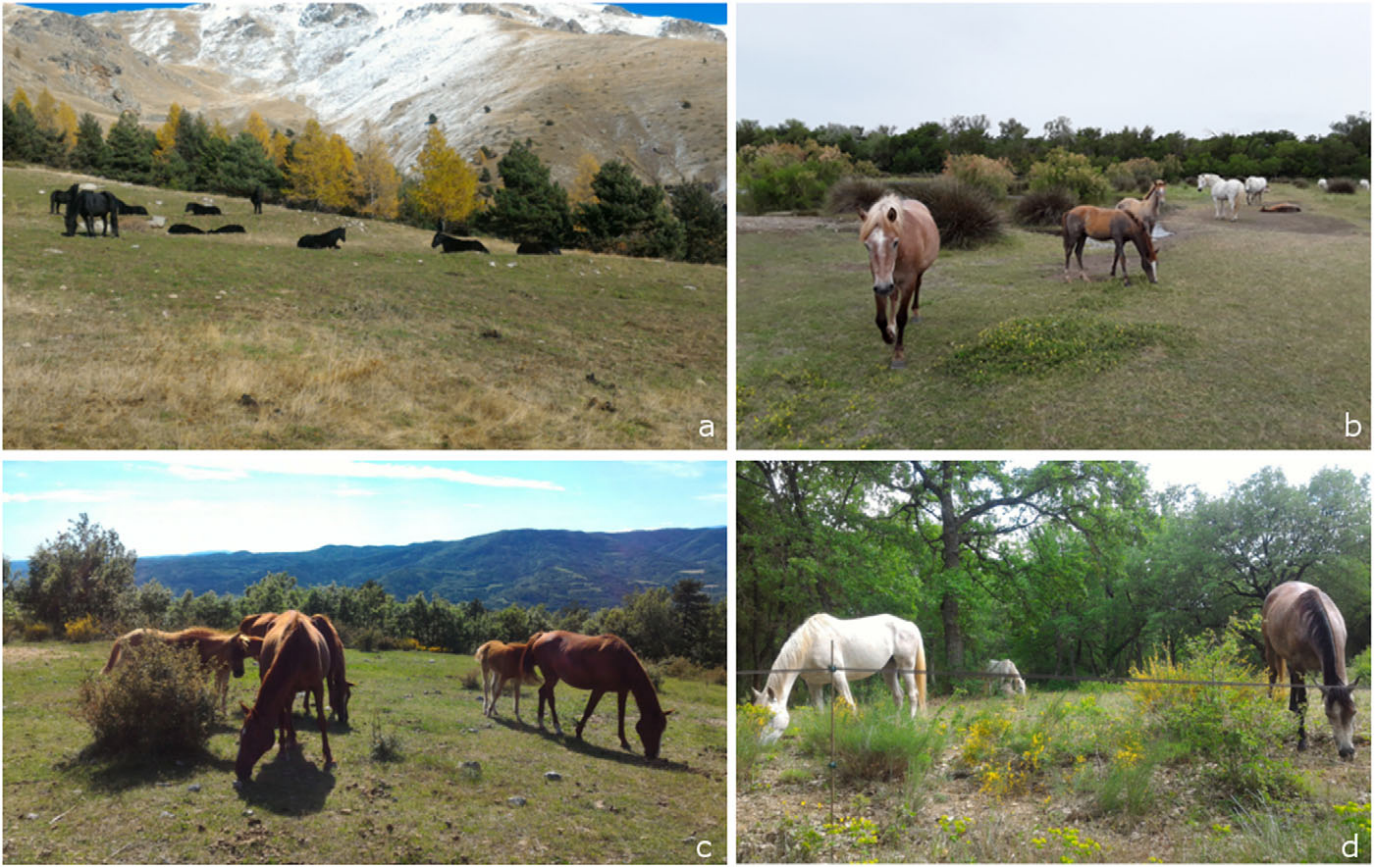
Example pictures of horses kept in “parcours”. a) Alpes Maritimes region; b) Cote d’azurregion, c) Provence region; d) Provence region.

‘Parcours’ are semi-natural areas grazed by domestic herbivores, such as horses. These areas have spontaneous and heterogeneous plant cover, with a low and very seasonal herbaceous production. They tend to prevail in difficult pedoclimatic environments (e.g. shallow soils, steep reliefs, frequent droughts), and are distinguishable by the degree of brushwood in lawns, moors and woods (Figure 1). In our study, herbaceous and woody plants provided a food resource that is heterogeneous in time and space. Grasses (e.g. brome, brachypod, dactyl) represented an important part of the horses’ diet. The horses consumed the green leaves with or without stems and ears, or only took the ears. The other herbaceous plants in the form of leaves or flowering stems were also widely consumed, whether legumes (e.g. vetch, clover) or others (e.g. thistle, yellow bedstraw, catananche). The horses also ate leaves and leafy branches of woody trees (e.g. beech, oak, service tree) and flowers (e.g. broom hispanica).

### Horses and facilities

Six farms in Région Sud Provence-Alpes-Côte d’Azur (France) were visited between June and November 2019. The following selection criteria for the facilities were employed: location in the Région Provence-Alpes-Cote d’Azur of France, adoption of an outdoor group-housing system such as a ‘parcours’ all-year-round, the keeping of at least ten horses aged older than one year. All the selected facilities were contacted via telephone and study participation was on a voluntary basis. On each farm, all horses older than one year were included, giving a total of 171 horses. Assessed animals had a mean (± SD) age of 8.95 (± 6.65), ranging from 1–25 years and consisting of both sexes (M = 61; F = 86; G = 61; Fpr = 10) and a variety of different breeds (Arabian: n =117, Anglo-Arabian: n = 1, Merens: n = 12, Camargue: n = 37, Not Assessed [NA]: n = 4) kept for different purposes (endurance: n = 102, leisure: n = 1, breeding: n = 52, retired: n = 6, NA: n = 10). Group sizes were variable (5.76 [± 3.62] individuals per group) as were areas utilised (from less than 1 ha to more than 500 ha; available space per horse ranging from 475 m^2^ per horse to more than 71,000 m^2^). A total of 33 groups were assessed, ten of which comprised both adult horses and foals (< one year old); welfare evaluation was not conducted on foals.

### Welfare assessment

The second level of the AWIN Welfare assessment protocol for horses (AWIN 2015) was adopted. To adapt the assessment protocol to the outdoor group-housing system, the assessment protocol was modified: a total of 22 animal-based indicators and four resource-based indicators was included (Table 1). A veterinarian, experienced in horse behaviour and welfare evaluation, and co-author of the AWIN welfare assessment protocol for horses, performed all the assessments; detailed information regarding the training of the assessor on the protocol are reported in Dalla Costa *et al*. (2017b). Horses were not restrained during evaluation and when it was not possible to touch the horse (e.g. avoidance reactions to Avoidance Distance test and/or Forced Human Approach test), the animal was evaluated from a maximum distance of 1 m. As for lameness evaluation, horses were observed during a spontaneous 10-m walk; if necessary, horses were gently encouraged to walk by the observer either vocally or by waving their arms. Detailed information regarding the welfare assessment (description, assessment and scoring methods of each welfare indicator) are reported in the AWIN welfare assessment protocol for horses (AWIN 2015), which is freely available: https://air.unimi.it/retrieve/handle/2434/269097/384836/AWINProtocolHorses.pdf.

**Table 1.** Welfare assessment protocol applied (modified from AWIN 2015).

Welfare principles	Welfare criteria	Welfare indicators
Good feeding	Appropriate nutrition	Body Condition Score Management based: forage availability
	Absence of prolonged thirst	Resource-based: clean water availability
Good housing	Comfort around resting	Resource-based: shelter availability, bedding, turnout time
	Thermal stress	Signs of cold stress (shivering, apathy, huddling) or hot stress (increased frequency/depth of respiration, flared nostrils, profuse sweating, apathy)*
Good health	Absence of physical injuries	Integument alterations, swollen joints, lameness, prolapse
	Absence of disease	Hair coat condition, discharges, abnormal breathing, coughing
	Absence of pain and pain induced by management procedures	Horse Grimace Scale (HGS), signs of hoof neglect, lesions at mouth corners
Appropriate behaviour	Expression of social behaviour	Positive and negative social interactions* Resource-based: possibility of social interaction
	Expression of other behaviours	Stereotypies
	Good human-animal relationship	Human-animal relationship tests (Avoidance Distance test, Forced Human Approach test)
	Positive emotional state	Qualitative Behavioural Assessment (QBA)*

* Results of these indicators are not presented in the paper.

### Statistical analysis

Data collected on-farm were compiled into an Excel file and subsequently descriptive statistics were performed; the proportion of satisfactory or unsatisfactory scores for each welfare indicator was calculated.

### Ethics

This study was conducted in compliance with Directive 2010/63/EU of the European Parliament and of the Council of 22 September 2010 on the protection of animals used for scientific purposes and followed the requirements of the International Society for Applied Ethology (ISAE). The study received approval from the Comité d’éthique de Val de Loire (number CE19-2020-1908-1). No animals underwent more than minimal distress and all procedures conformed to a routine assessment as in good farm practices. Written informed consent was gained from the farmers prior to taking part in this research.

## Result and discussion

The results of the welfare assessment will be reported and discussed for each welfare principle (‘Good feeding’, ‘Good housing’, ‘Good health’ and ‘Appropriate behaviour’).

### Good feeding

**Figure 2.**
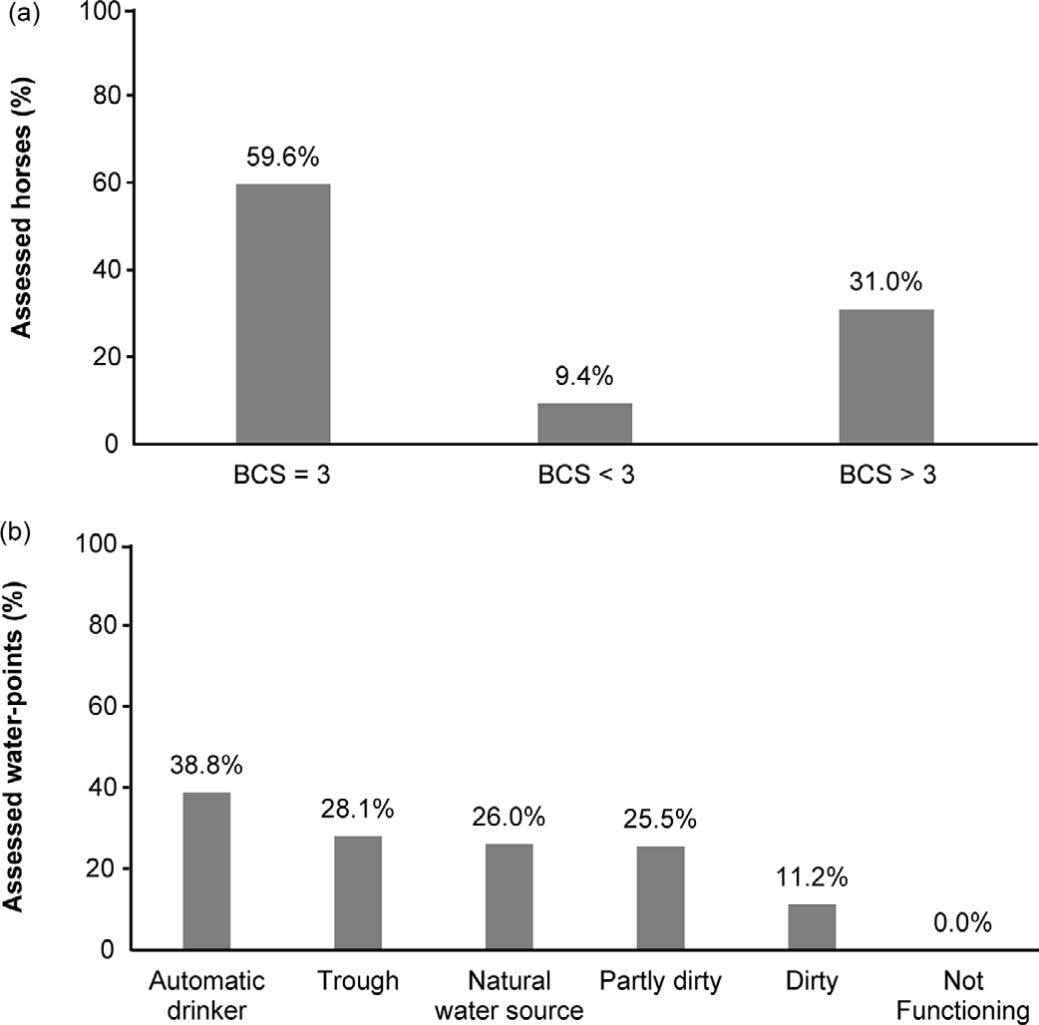
Results of the welfare assessment (% of horses) related to the principle “goodfeeding” in parcour horses. a) Body condition score on a 5 point scale (AWIN 2015); b) water availability: type of waterpoint(automatic drinker, trough, natural water source); cleanliness of water point(partially dirty: water point dirty but water clean; dirty: water point andwater dirty) (AWIN 2015).

As regards the principle ‘Good feeding’ (Figure 2[a]), most of the assessed animals benefitted from appropriate nutrition (Body Condition Score [BCS] = 3; 59.6%). Extremes were rare (BCS = 1; 1.17% and BCS = 5; 1.17%). While not having a BCS of 3, most of the horses were over- (BCS > 3; 31%), rather than underweight (BCS < 3; 9.4%). Most of the overweight horses had a BCS = 4 (29.82%). Our results are in line with what has been previously observed in single-box-housed horses (Visser *et al*. 2014; Dalla Costa *et al*. 2017b), suggesting that group-housing in semi-extensive conditions such as ‘parcours’ does not represent a risk factor for poor nutrition. This result confirms what was suggested by Souris *et al*. (2007), who observed that horses released in a natural environment with temperate climate are able to adapt their daily intake according to pasture availability and changes to climate, maintaining a good BCS or improving it. However, as reported by Dalla Costa *et al*. (2014b) “excellent body condition in a horse does not necessarily mean that foraging need is fulfilled”, which is not the case in group-housing at pasture. In fact, this housing condition allows horses to express natural grazing behaviour, satisfying the behavioural need to forage (Ninomiya *et al*. 2004). The restriction of this behavioural pattern and the reduced time dedicated to feeding imposed by box-housing are considered risk factors for stereotypies (for a review, see Sarrafchi & Blokhuis 2013) and colic development (Hudson *et al*. 2001). While avoiding the risk of under-nourishing horses, it is important to keep in mind that excessive body fat is related both to health problems (such as insulin resistance, colic, laminitis) and loss of performance (Geor & Acvim 2008; Carter *et al*. 2009; Becvarova & Pleasant 2012; Galantino-Homer & Engiles 2012). To maintain an appropriate bodyweight, horses need a daily intake of their bodyweight in dry matter of forage and are readily able to match or even exceed their required daily dry matter intake with 24-h access to good quality pasture (Nadeau 2006; Dowler *et al*. 2012; Siciliano 2012; Fiorellino *et al*. 2014). Five out of six farms in our study also provided horses with access to hay, in addition to pasture. This may have contributed to the high percentage of overweight horses in our sample. Owners may wish to supply hay to guarantee an adequate food intake; however, when grazing is permitted, this supplementation may put the animal at unnecessary risk of increasing weight. On the other hand, it was noticeable that the overweight horses were mostly Camargue and Merens. These are rustic breeds renowned for valuing their food very highly. Therefore, the natural and fodder resources provided on the ‘parcours’ seem excessively rich for some of these animals. Moreover, none of the farmers restricted the amount of pasture available to horses on a daily basis; a management trait that puts horses at risk of excessive weight gain (Dowler *et al*. 2012; Siciliano 2012).

Horses had free access to a water-point (Figure 2[b]), which consisted of an automatic drinker (38.78%), a trough (28.06%) or a natural source of water (26.02%). In 7.14% of cases it was not possible to find and check the water-point, probably as a result of the size of the pasture in question. When a water-point was available, 27.55% of horses had access to clean water, while 25.51% had access to a partially dirty water-point (water-point dirty but water clean) and 11.22% to a dirty one (water-point and water dirty). Dalla Costa and colleagues (2017b) found similar results: the drinkers of single-housed horses were partially dirty (24.5%) and dirty (17.5%). Water supply is a recognised issue in other farm animals kept on pasture with problems including short periods of water availability, water present only in certain areas where animals are grazing, absence of a man-made water supply and algal contamination depending on temperature and light (Kamphues & Ratert 2014). Moreover, the daily inspection and cleaning of water-points in very large pastures can present a challenge which may explain the numbers of partially dirty and dirty water-points found in the present study. Another important aspect is drinkability and accessibility of water-points, especially when the only source of water is a natural one. Water quality, in such cases, should be checked, to ensure appropriate standards of drinkability, i.e. chemical, physical, and biological characteristics (Kamphues & Ratert 2014). Cleanliness of water is of paramount importance, since horses are known to refuse dirty water (Friend 2000); furthermore, water troughs and buckets should be cleaned regularly since shared water sources are a common source of disease (Lardy *et al*. 2008). Another aspect to take into account when considering horses kept on pasture is the water temperature in the trough: both cold water in winter and warm water in summer can lead to a decrease in water consumption (Kristula & Mcdonnell 1994), which is reportedly the primary predisposing factor for impaction colic (Kaya *et al*. 2009).

### Good housing

**Figure 3.**
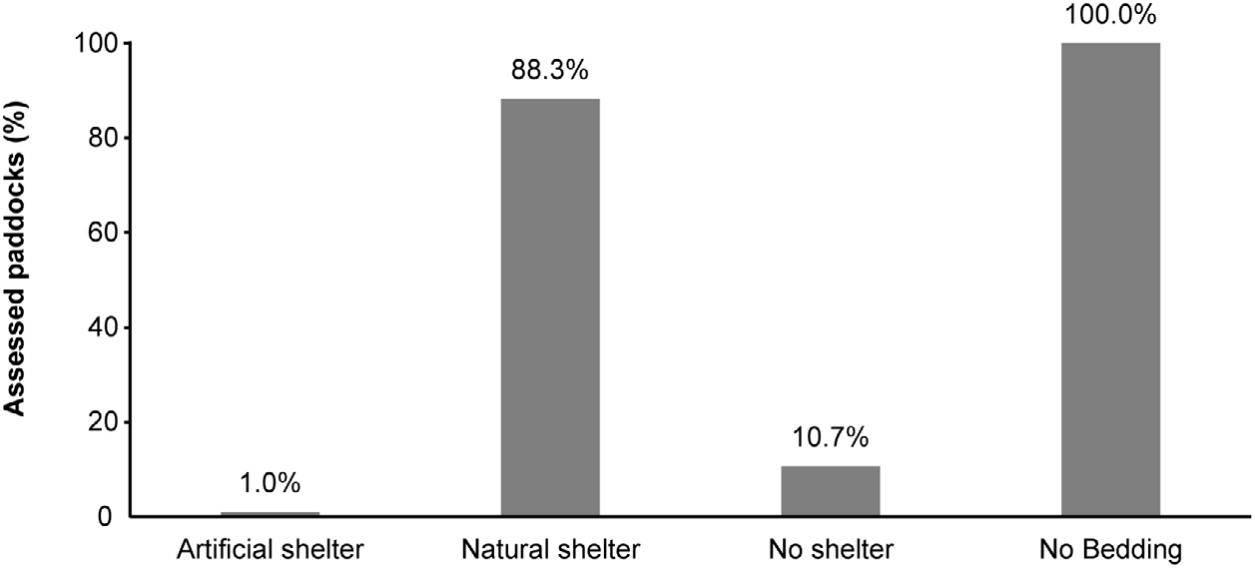
Results of the welfare assessment (% of horses) related to the principle “Good housing”(shelter availability and bedding) in parcour horses.

As regards the principle ‘Good housing’, all of the horses being evaluated were able to move freely throughout the entire day. For horses, movement is a highly motivated behaviour (Chaplin & Gretgrix 2010) and restrictions placed on it are known to impact upon their welfare (McGreevy *et al*. 1995; Cooper & Albentosa 2005). One possible concern regarding horses kept on pasture is their ability to shelter during inclement weather (Snoeks *et al*. 2015). In our sample, 10.7% of horses had no access to a shelter (Figure 3) and such an absence represents a considerable risk factor for horse welfare: the thermo-neutral zone for horses is estimated to lie within the range of 5 to 25°C (Morgan 1998) and when the environmental temperature deviates from this range, thermoregulation is achieved through changes in behaviour, including shelter-seeking (Cymbaluk 1994; Snoeks *et al*. 2015). Several studies have demonstrated the need for horses to be able to access shelter during rainy or windy days (Tyler 1972; Duncan 1985; Autio & Heiskanen 2005; Mejdell & Bøe 2005; Ingólfsdóttir & Sigurjónsdóttir 2008). Shelter-seeking is also observed on hot, sunny days (Heleski & Murtazashvili 2010; Holcomb *et al*. 2014). Thermoregulation may not always be the main motivating factor for seeking shelter: horses also prefer to use a shelter to alleviate harassment from insects (Keiper & Berger 1982; Gòrecka-Bruzda & Jezieski 2007). The majority of horses in the present study had access to a shelter: natural, such as trees (83.3%), or artificial (1%). Although one publication (Snoeks *et al*. 2015) reported, given a choice, horses preferred artificial shelters over natural ones, especially during cold and rainy conditions, artificial shelters are difficult to provide when horses move over large areas as was the case in our study. It should also be taken into consideration that non-artificial shelters are more in keeping with the natural environment and that when only natural shelters are available, horses prefer to spend time under dense vegetation (Pratt *et al*. 2016).

None of the visited farms used bedding (Figure 3), it may be hypothesised that the owners of horses kept on ‘parcours’ did not consider it necessary to provide bedding, since horses were able to choose a more comfortable spot upon which to lie down. It is worth noting that bed sores were not observed (see *Good health*).

### Good health

**Figure 4.**
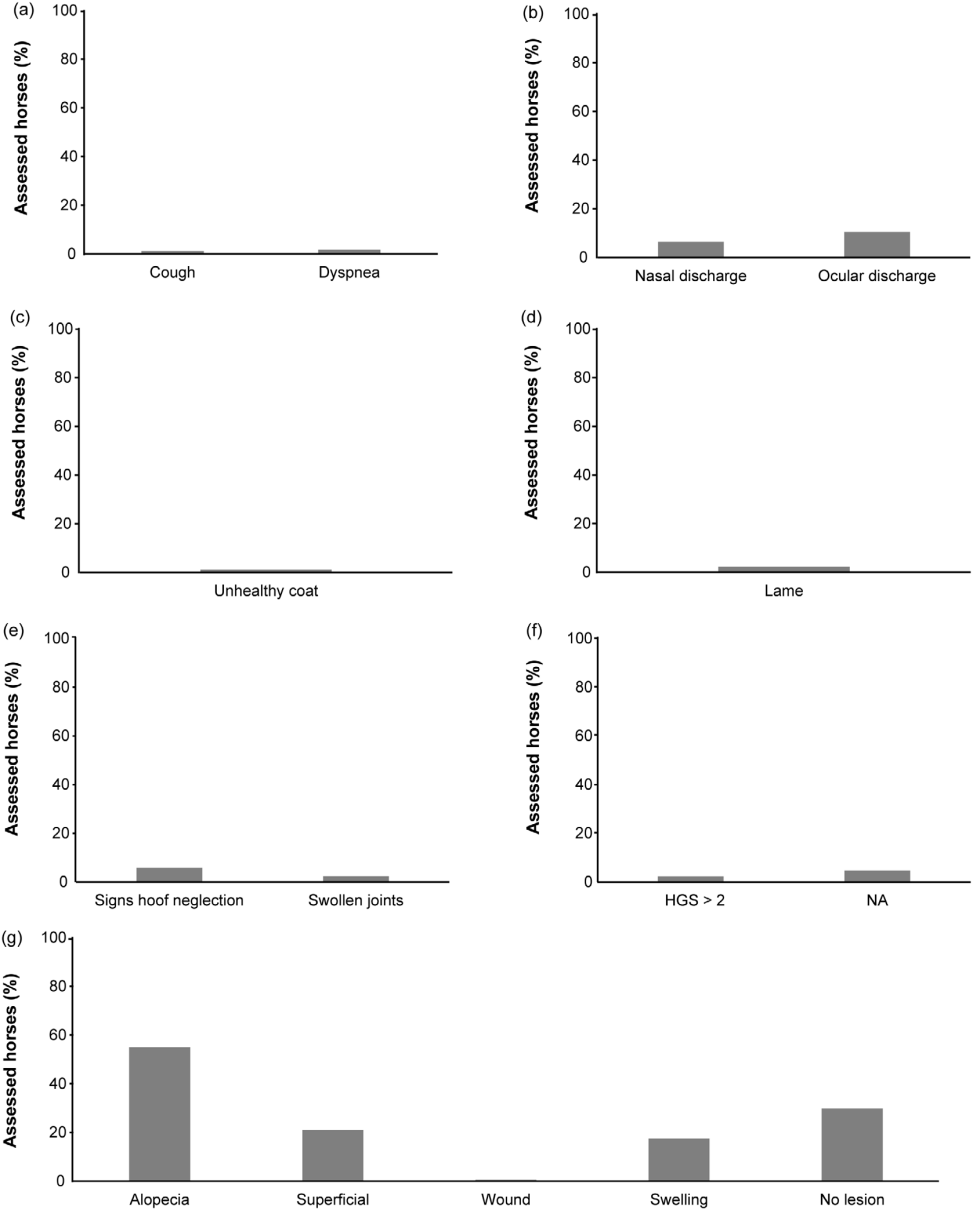
Results of the welfare assessment (%of horses) related to the principle “good health” in parcour horses. a) cough and dyspnoea; b) nasal and ocular discharges; c) coat condition; d) lameness; e) signs of hoof neglect andswollen joints; f) HGS; g) skin lesions.

The studied horses generally benefitted from good health and none presented any severe health conditions (Figure 4). While we did not perform any clinical examination on assessed horses, indicators such as coughing, abnormal breathing, nasal and ocular discharge were chosen since they are well recognised symptoms of a diverse variety of respiratory problems (Halliwell *et al*. 1993). These are reported to be common in horses kept in single boxes with a prevalence ranging between < 3 and 16.9% (Wheeler *et al*. 2002; Hotchkiss *et al*. 2007; Visser *et al*. 2014). In ‘parcours’ housed horses, we found that 1.8% (3 out of 171) showed dyspnoea, 1.2% (2 out of 171) coughing, and 6.4% (11 out of 171) clear, serous nasal discharge, while none presented purulent or haematic discharge (Figure 3[a] and [b]). This prevalence is higher than found by Dalla Costa *et al*. (2017b), but lower than has been reported for stabled horses (Wheeler *et al*. 2002; Hotchkiss *et al*. 2007; Visser *et al*. 2014). Since respiratory problems have been associated with the housing system, stable hygiene practices and bedding choice (Clarke 1987; Halliwell *et al*. 1993), our results can perhaps be attributed to low ammonia levels, dust concentration and fungal presence in an open-air environment. Similarly, a low prevalence of ocular discharge was observed (10.5% of horses; 18 out of 171) and no individuals showed a thick, purulent or haematic discharge. Visser *et al*. (2014) reported a prevalence of 20% of ocular discharge in stabled horses and their risk factors for this were the number of horses housed in the same stable and the absence of a viable air outlet.

Lameness is generally considered to be a common cause of welfare impairment in horses and in stabled horses the reported prevalence ranges from 13 to 33% (Murray *et al*. 2010; Ireland *et al*. 2012; Lesimple *et al*. 2012; Visser *et al*. 2014). In the present study, a much lower percentage was identified (2.3%) (Figure 4[d]). The cause of lameness was not investigated, but it is worth noting that one horse also showed swollen joints and three showed varying degrees of hoof neglect, which can be responsible for lameness. Several risk factors have been described for lameness: age (older horses are at greater risk of lameness), current use of horse (riding school use or recreation increases lameness risk), back pain caused by inappropriate saddle, foot problems, training regimen (using only one surface for training increases the risk of lameness) (Cooper & Albentosa 2005; Murray *et al*. 2010; Visser *et al*. 2014). It could be hypothesised that horses kept in ‘parcours’, having greater scope to exercise freely on different grounds, developed a musculoskeletal system better adapted to a range of surfaces and exercises, consequently reducing the risk of injuries during sports activities. Murray *et al*. (2010) also identified the lack of warm-up before exercise as a risk factor for musculoskeletal injuries and subsequent lameness. Horses kept on ‘parcours’, permanently able to move, could perform a ‘natural warm-up’, decreasing the risk of lameness.

In the present study only 29.8% of horses presented an intact skin (Figure 4[g]). It is worth noting that most of the horses (53.8%) showed areas of alopecia, while 23.4% presented superficial lesions and 0.6% deep wounds (Figure 4[g]). These results differ from those of Dalla Costa and colleagues (2017b) on single-housed horses, where the majority of horses had no skin lesions. The causes of skin lesions were not investigated, but alopecia is often related to itch which may be caused by insect bites, ectoparasites or allergic reactions. In fact, 18.8% of horses showed swellings on the skin, probably related to insect biting. Fly control at pasture may represent a challenge for owners; some management practices (such as the use of repellents, fly traps, protective masks and/or rugs, and the frequent removal of dung) may help in reducing fly bites (Gòrecka-Bruzda & Jezieski 2007). It should, however, be noted that only a limited number of products are currently approved for treatment of ectoparasites in horses, meaning that these products should be used judiciously with special emphasis on the safety of these products for horses, people and the environment (Karasek *et al*. 2020). Superficial lesions may be caused by scratches from branches or rocks, or through aggressive interactions with other horses. One of the major concerns preventing owners from keeping their horses in a group is the possibility of aggressive behaviours causing lesions or restricted access to crucial resources (McGreevy 2004). However, several studies have demonstrated that the level of aggression significantly decreases with increased group stability (van Dierendonck *et al*. 2004; Hartmann *et al*. 2012; Sigurjónsdóttir & Haraldsson 2019) and with increased area availability per horse (up to at least 300 m^2^) (Flauger & Krueger 2013). Indeed, group stability, as with feral horses (Waring 2003; Stanley *et al*. 2018), allows stable dominance relationships and friendship networks, reducing numbers of aggressive interactions among members (Sigurjónsdóttir *et al*. 2003; van Dierendonck *et al*. 2004; Fureix *et al*. 2012; Granquist *et al*. 2012; Hartmann *et al*. 2012). Equally, recurrent changes in group composition sees a rise in the number of interactions, mainly agonistic ones (Hartmann *et al*. 2009; Fureix *et al*. 2012). This is explained by the fact that in stable groups each individual is aware of the social network and, consequently, the aggression is ritualised (Rutberg & Greenberg 1990; Heitor *et al*. 2006; Hartmann *et al*. 2012). Therefore, the risk factor for injuries should not be considered a result of the group housing *per se*, but more the lack of group *stability* (Fureix *et al*. 2012). In our study, the mean number of aggressive interactions per horse per hour was 0.76 (± 1.2) (data not shown) which seems somewhat low compared to data reported for horses observed in semi-natural conditions (Flauger & Krueger 2013) and the average area per horse was much greater than 300 m^2^. Therefore, these two factors do not seem to be the main reasons for the large proportion of observed skin lesions.

It could be hypothesised that, compared to owners who keep their horses in boxes, owners favouring horses at pasture are perhaps less attentive to hoof care, especially when horses are not used on a daily basis for sport activities. However, only 5.8% of horses (ten out of 171) in our sample presented some degree of hoof neglect (Figure 3[e]); a result comparable with the prevalence reported elsewhere (Dalla Costa *et al*. 2017b). Regardless of housing system, daily care and regular routine farriery are fundamental since neglect of these practices predisposes to the development of foot problems (Kummer *et al*. 2006; van Eps 2012; Leśniak *et al*. 2017).

The Horse Grimace Scale (HGS) is a facial-expression-based pain coding system (Dalla Costa *et al*. 2014a) which can be considered a specific tool to assess pain in horses (Dalla Costa *et al*. 2017a) and easily applicable by non-expert observers (Dai *et al*. 2020). An HGS value⩾2 is considered an indicator of pain (Dalla Costa *et al*. 2018). In the present study, the HGS score was ⩾2 in 2.3% of cases (Figure 4[f]), this is similar to horses kept in single boxes (Dalla Costa *et al*. 2017b); thus, confirming that horses were regularly checked for possible pain-related conditions. It is important to underline that in 4.6% of cases, it was not possible to assess HGS, meaning that horses were not close enough to permit an accurate scoring or were wearing masks partly covering their head.

### Appropriate behaviour

**Figure 5.**
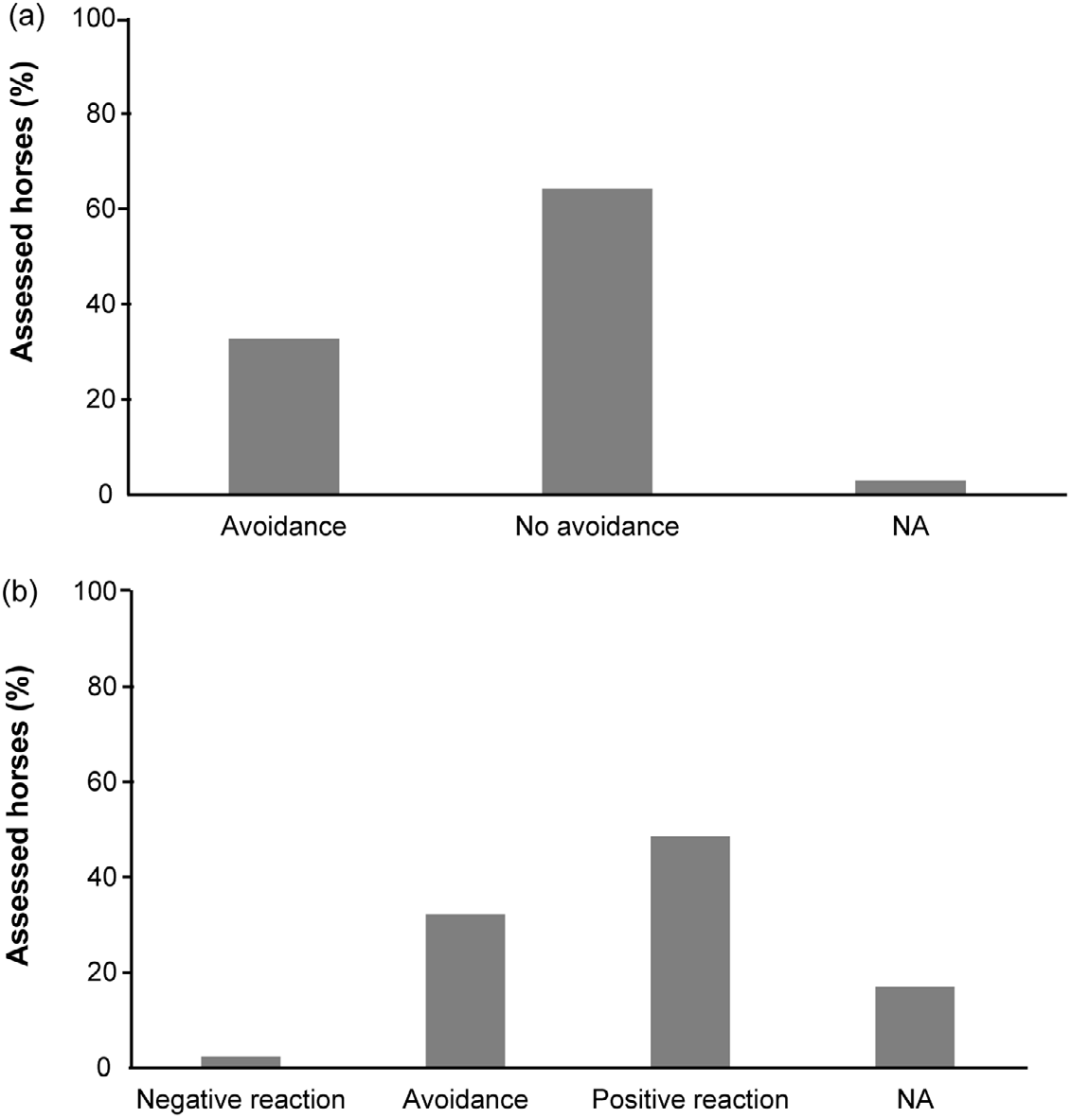
Results of the welfare assessment (% of horses) related to the principle“appropriate behaviour” in parcour horses. a) Avoidance Distance test; b)Forced Human Approach test.

Figure 5 reports results regarding the principle ‘Appropriate behaviour.’ Regarding social interaction, horses were kept in groups of different dimensions (mean 5.76 [± 3.62] conspecifics); ten groups included foals and nine included stallions. Only one stallion (0.6%), used for reproduction, was kept alone; visual and olfactory contact with other horses were possible for this individual. In both Italy and Germany it was reported that 22.3% of stabled horses had no scope for visual or physical contact (Dalla Costa *et al*. 2017b). Hockenhull and Creighton (2015) reported that in the UK 3% of horses face the same situation. As horses are a social species, social interaction with conspecifics is a behavioural need. The restriction imposed by housing conditions is deemed responsible for the development of a range of abnormal behaviours, such as stereotypies (McGreevy *et al*. 1995; Cooper & Albentosa 2005). Previous studies reported a prevalence of stereotypies in horses kept in single boxes ranging from 14.4 (Ruet *et al*. 2019) to 32.5% (McGreevy *et al*. 1995), while in the present study we observed only 1.2% of stereotypies (two horses out of 171). A recognised risk factor for stereotypy development is the frustration of fundamental needs (e.g. grazing, movement, social relationship) (McGreevy *et al*. 1995; Cooper & Albentosa 2005); being housed in groups and having permanent access to pasture can therefore explain this low prevalence of stereotypies observed here.

A possible concern preventing owners from keeping their horses at pasture is the difficulty of catching them (McGreevy 2004). Counter-intuitively, 64.3% of horses in our sample showed no avoidance reactions when approached by the unknown assessor, similarly to the box-housed horses included in the AWIN population (Figure 5[a]). Moreover, 48.5% of horses showed positive responses to the Forced Human Approach (FHA) test (Figure 5[b]). In the FHA test, 32.2% of horses showed avoidance reaction, only 2.3% showed some aggressive behaviours; for 17% of horses it was not possible to perform the FHA test, because they went away from the observer during the Avoidance Distance (AD) test (Figure 5[b]). In a previous study on horses kept in single boxes, a larger proportion exhibited positive reactions to the AD test (Dalla Costa *et al*. 2017b); however, it is worth pointing out that the horses included in the present study preferred to move away from the observer, when unwilling to interact, instead of being aggressive, thus potentially reducing the risks for human injuries. Similar results were obtained in a previous study comparing two groups of ponies kept in a group on pasture or housed in individual boxes, in restricted conditions (Dany *et al*. 2017). Avoidance from an undesired stimulus is a natural behaviour for a prey species and suggests that observed horses had the perception of being able to control their own environment, deciding when to interact instead of feeling forced to do so with humans. Interactions with owners were not formally noted, however the assessor observed that most of the horses were more friendly with the owner and showed avoidance reactions less frequently. To overcome the catching difficulties potentially perceived by owners (McGreevy 2004), specific training to teach the horse to come when called using non aversive methods may be useful (Sankey *et al*. 2010). Training could also help in simplifying horses’ daily inspections.

### Animal welfare implications

Outdoor group housing could be seen as having more similarities with feral horse living conditions, however it is considered to increase the risk of developing injury and illness. This study reports, for the first time, results from a comprehensive welfare data collection carried out on group-housed horses on ‘parcours.’ The reported outcomes can help in creating a common database on horse welfare status and understanding underlying relations with housing conditions and management.

## Conclusion

The application of a complete and comprehensive assessment method to evaluate the welfare of group-housed horses kept on ‘parcours’ proved to be feasible and useful in identifying areas of practice that can be linked to good welfare and areas where improvements are required. The findings showed that horses kept on ‘parcours’ presented few abnormal behaviours such as stereotypies, could move freely for most of the day and interact with conspecifics, at the same time maintaining a good relationship with humans. The main welfare concerns were related to the availability of water sources, lack of artificial shelters and presence of superficial integument alterations such as alopecia, probably linked to sub-optimal control of external parasites. Excessive weight gain was observed in a significant proportion of horses (especially in those facilities where hay was administered in addition to natural resources). Study limitations are mainly represented by the relatively small number of facilities involved in this study, especially in terms of geographical location, thus the sample may not accurately reflect the welfare status of all the horses kept on ‘parcours’ or at pasture. Stronger conclusions could be derived from a direct comparison, adopting an inferential statistical approach, on data collected using the same welfare assessment method on horses kept under different management systems in similar geographical locations. Following the same approach, further harmonised data collection is required to enlarge the sample and perhaps include other housing conditions.

## References

[r1] Auriane Hurtes 2015 A survey on the feeding of competition horses and perceptions of forage in the UK and Sweden. Swedish University of Agricultural Sciences: Uppsala, Sweden.

[r2] Autio E and Heiskanen M 2005 Foal behaviour in a loose housing/paddock environment during winter. Applied Animal Behaviour Science 91: 277–288. 10.1016/j.applanim.2004.10.012

[r3] AWIN 2015 *AWIN welfare assessment protocol for horses.* 10.13130/AWIN_horses_2015

[r4] Becvarova I and Pleasant IS 2012 Managing obesity in pasture-based Horses. Compendium: Continuing Education for Veterinarians 34: E1–E4. 22488595

[r5] Carter RA , Treiber KH , Geor RJ , Douglass L and Harris PA 2009 Prediction of incipient pasture-associated laminitis from hyperinsulinaemia, hyperleptinaemia and generalised and localised obesity in a cohort of ponies. Equine Veterinary Journal 41: 171–178. 10.2746/042516408X342975 19418747

[r6] Chaplin SJ and Gretgrix L 2010 Effect of housing conditions on activity and lying behaviour of horses. Animal 4: 792–795. 10.1017/S1751731109991704 22444135

[r7] Clarke AF 1987 A review of environmental and host factors in relation to equine respiratory disease. Equine Veterinary Journal 19: 435–441. 10.1111/j.2042-3306.1987.tb02638.x 3315647

[r8] Cooper J and Albentosa M 2005 Behavioural adaptation in the domestic horse: potential role of apparently abnormal responses including stereotypic behavior. Livestock Production Science 92: 117–182. 10.1016/j.livprodsci.2004.11.017

[r9] Cymbaluk NF 1994 Thermoregulation of horses in cold, winter weather: a review. Livestock Production Science 40: 65–71. 10.1016/0301-6226(94)90266-6

[r10] Dai F , Leach M , Macrae AM , Minero M and Costa ED 2020 Does thirty-minute standardised training improve the inter-observer reliability of the horse grimace scale (HGS)? A case study. Animals 10: 1–7. 10.3390/ani10050781 PMC7277819 32365927

[r11] Dalla Costa E , Bracci D , Dai F , Lebelt D and Minero M 2017a Do different emotional states affect the Horse Grimace Scale score? A pilot study. Journal of Equine Veterinary Science 54: 114–117. 10.1016/j.jevs.2017.03.221

[r12] Dalla Costa E , Dai F , Lebelt D , Scholz P , Barbieri S , Canali E and Minero M 2017b Initial outcomes of a harmonized approach to collect welfare data in sport and leisure horses. Animal 11: 254–260. 10.1017/S1751731116001452 27406177

[r13] Dalla Costa E , Dai F , Lebelt D , Scholz P , Barbieri S , Canali E , Zanella AJ and Minero M 2016 Welfare assessment of horses: the AWIN approach. Animal Welfare 25: 481–488. 10.7120/09627286.25.4.481

[r14] Dalla Costa E , Minero M , Lebelt D , Stucke D , Canali E and Leach MC 2014a Development of the Horse Grimace Scale (HGS) as a pain assessment tool in horses undergoing routine castration. PLoS One 9: e92281. 10.1371/journal.pone.0092281 PMC3960217 24647606

[r15] Dalla Costa E , Murray LMA , Dai F , Canali E and Minero M 2014b Equine on-farm welfare assessment: A review of animal-based indicators. Animal Welfare 23: 323–341. 10.7120/09627286.23.3.323

[r16] Dalla Costa E , Pascuzzo R , Leach MC , Dai F , Lebelt D , Vantini S and Minero M 2018 Can grimace scales estimate the pain status in horses and mice? A statistical approach to identify a classifier. PLoS One 13: e0200339. 10.1371/journal.pone.0200339 PMC6070187 30067759

[r17] Dany P , Vidament M , Yvon J , Reigner F , Barrière P , Riou M , Layne A , Lansade L , Minero M , Dalla Costa E and Briant C 2017 Protocole d’évaluation du bien être chez le cheval ‘AWIN Horse’: essai en conditions expérimentales et premières évaluations sur le terrain. 43th Journée de la Recherche Equine pp 159–162. 16 March 2017, Paris, France. [Title Translation: AWIN welfare assessment protocol for horses: test under experimental conditions and first evaluations in the field]

[r18] Dowler LE , Siciliano PD , Pratt-Phillips SE and Poore M 2012 Determination of pasture dry matter intake rates in different seasons and their application in grazing management. Journal of Equine Veterinary Science 32: 85–92. 10.1016/j.jevs.2011.06.006

[r19] Duncan P 1985 Time-budgets of Camargue horses III. Environmental influences. *Behaviour* 92: 188–208. 10.1163/156853985X00442

[r20] EFSA Panel on Animal Health and Welfare (AHAW) 2012 Statement on the use of animal-based measures to assess the welfare of animals. EFSA Journal 10(6): 2767. 10.2903/j.efsa.2012.2767.

[r21] Fiorellino NM , McGrath JM , Momen B , Kariuki SK , Calkins MJ and Burk AO 2014 Use of best management practices and pasture and soil quality on maryland horse farms. Journal of Equine Veterinary Science 34: 257–264. 10.1016/j.jevs.2013.05.009

[r22] Flauger B and Krueger K 2013 Aggression level and enclosure size in horses (*Equus caballus*). Pferdeheilkunde 29: 495–504.

[r23] Foster CN , Banks SC , Cary GJ , Johnson CN , Lindenmayer DB and Valentine LE 2020 Animals as agents in fire regimes. Trends in Ecology and Evolution 35: 346–356. 10.1016/j.tree.2020.01.002 32187509

[r24] Fraser D 2008 Understanding animal welfare. Acta Veterinaria Scandinavica 50: 1–7. 10.1186/1751-0147-50-S1-S1 19049678 PMC4235121

[r25] Fraser D 2009 Animal behaviour, animal welfare and the scientific study of affect. Applied Animal Behaviour Science 118: 108–117. 10.1016/j.applanim.2009.02.020

[r26] Friend T 2000 Dehydration, stress, and water consumption of horses during long-distance commercial transport. Journal of Animal Science 78: 2568–2580. 10.2527/2000.78102568x 11048922

[r27] Fureix C , Bourjade M , Henry S , Sankey C and Hausberger M 2012 Exploring aggression regulation in managed groups of horses *Equus caballus* . Applied Animal Behaviour Science 138: 216–228. 10.1016/j.applanim.2012.02.009

[r28] Galantino-Homer HL and Engiles JB 2012 Insulin resistance and laminitis in broodmares. Journal of Equine Veterinary Science 32: 680–688. 10.1016/j.jevs.2012.08.220

[r29] Gehlen H , Krumbach K and Thöne-Reineke C 2021 Keeping stallions in groups: Species-appropriate or relevant to animal welfare? Animals 11: 1–11. 10.3390/ani11051317 PMC8147931 34064522

[r30] Geor RJ and Acvim D 2008 Metabolic predispositions to laminitis in horses and ponies: Obesity, insulin resistance and metabolic syndromes. Journal of Equine Veterinary Science 28: 753–759. 10.1016/j.jevs.2008.10.016

[r31] Gmel AI , Zollinger A , Wyss C , Bachmann I and Freymond SB 2022 Social box: Influence of a new housing system on the social interactions of stallions when driven in pairs. Animals 12: 1–13. 10.3390/ani12091077 PMC9099530 35565503

[r32] Gòrecka-Bruzda A and Jezieski T 2007 Protective behaviour of Konik horses in response to insect harassment. Animal Welfare 16: 281–283.

[r33] Granquist SM , Gudrun A and Sigurjonsdottir H 2012 The effect of stallions on social interactions in domestic and semi feral harems. Applied Animal Behaviour Science 141: 49–56. 10.1016/j.applanim.2012.07.001

[r34] Halliwell REW , McGorum BC , Irving P and Dixon PM 1993 Local and systemic antibody production in horses affected with chronic obstructive pulmonary disease I. Veterinary Immunology and Immunopathology 38: 201–215. 10.1016/0165-2427(93)90081-E 8291200

[r35] Hampson BA , Laat MADE , Mills PC and Pollitt CC 2010a Distances travelled by feral horses in ‘outback ’ Australia 42: 582–586. 10.1111/j.2042-3306.2010.00203.x 21059064

[r36] Hampson BA , Morton JM , Mills PC , Trotter MG , Lamb DW and Pollitt CC 2010b Monitoring distances travelled by horses using GPS tracking collars. Australian Veterinary Journal 88: 176–181. 10.1111/j.1751-0813.2010.00564.x 20529024

[r37] Hartmann E , Christensen JW and Keeling LJ 2009 Social interactions of unfamiliar horses during paired encounters: Effect of pre-exposure on aggression level and so risk of injury. Applied Animal Behaviour Science 121: 214–221. 10.1016/j.applanim.2009.10.004

[r38] Hartmann E , Søndergaard E and Keeling LJ 2012 Keeping horses in groups: A review. Applied Animal Behaviour Science 136: 77–87. 10.1016/j.applanim.2011.10.004

[r39] Heitor F , do Mar Oom M and Vincente L 2006 Social relationships in a herd of Sorraia horses Part II. Factors affecting affiliative relationships and sexual behaviours. Behavioural Processes 73: 231–239. 10.1016/j.beproc.2006.05.005 16828984

[r40] Heleski CR and Murtazashvili I 2010 Daytime shelter-seeking behavior in domestic horses. Journal of Veterinary Behavior: Clinical Applications and Research 5: 276–282. 10.1016/j.jveb.2010.01.003

[r41] Hockenhull J and Creighton E 2015 The day-to-day management of UK leisure horses and the prevalence of owner-reported stable-related and handling behaviour problems. Animal Welfare 24: 29–36. 10.7120/09627286.24.1.029

[r42] Hoffman CJ , Costa LR and Freeman LM 2009 Survey of feeding practices, supplement use, and knowledge of equine nutrition among a subpopulation of horse owners in New England. Journal of Equine Veterinary Science 29: 719–726. 10.1016/j.jevs.2009.08.005

[r43] Holcomb K , Tucker C and Stull C 2014 Preference of domestic horses for shade in a hot, sunny environment. Journal of animal science 92: 1708–1717. 10.2527/jas2013-7386 24492578

[r44] Hotchkiss JW , Reid SWJ and Christley RM 2007 A survey of horse owners in Great Britain regarding horses in their care. Part 1: Horse demographic characteristics and management. Equine Veterinary Journal 39: 294–300. 10.2746/042516407X177538 17722719

[r45] Hudson JM , Cohen ND , Gibbs PG and Thompson JA 2001 Feeding practices associated with colic in horses. Journal of the American Veterinary Medical Association 219: 1419–1425. 10.2460/javma.2001.219.1419 11724182

[r46] Ingólfsdóttir HB and Sigurjónsdóttir H 2008 The benefits of high rank in the wintertime — A study of the Icelandic horse. Applied Animal Behaviour Science 114: 485–491. 10.1016/j.applanim.2008.04.014

[r47] Ireland J , Clegg P , Mcgowan C , McKane S , Chandler K and Pinchbeck G 2012 Comparison of owner-reported health problems with veterinary assessment of geriatric horses in the United Kingdom. Equine Veterinary Journal 44: 94–100. 10.1111/j.2042-3306.2011.00394.x 21696434

[r48] Jansson A and Harris PA 2013 A bibliometric review on nutrition of the exercising horse from 1970 to 2010. Comparative Exercise Physiology 9: 169–180. 10.3920/CEP13018

[r49] Kamphues J and Ratert C 2014 Drinking water quality in food producing animals. Proceedings of International Workshop: New updates in Animal Nutrition, Natural Feeding Sources and Environmental Sustainability pp 5–13. Arzachena, Italy.

[r50] Karasek I , Butler C , Baynes R and Werners A 2020 A review on the treatment and control of ectoparasite infestations in equids. Journal of Veterinary Pharmacology and Therapeutics 00: 1–8. 10.1111/jvp.12874 32488977

[r51] Kaya G , Sommerfeld-Stur I and Iben C 2009 Risk factors of colic in horses in Austria. Journal of Animal Physiology and Animal Nutrition 93: 339–349. 10.1111/j.1439-0396.2008.00874.x 19646108

[r52] Kaya-Karasu G , Huntington P , Iben C and Murray JA 2018 Feeding and management practices for racehorses in Turkey. Journal of Equine Veterinary Science 61: 108–113. 10.1016/j.jevs.2017.04.009

[r53] Keiper RR and Berger J 1982 Refuge-seeking and pest avoidance by feral horses in desert and island environments. Applied Animal Ethology 9: 111–120. 10.1016/0304-3762(82)90187-0

[r54] Kristula MA and McDonnell SM 1994 Drinking water temperature affects consumption of water during cold weather in ponies. Applied Animal Behaviour Science 41: 155–160. 10.1016/0168-1591(94)90020-5

[r55] Kummer M , Geyer H , Imboden I , Auer J and Lischer C 2006 The effect of hoof trimming on radiographic measurements of the front feet of normal Warmblood horses. Veterinary Journal 172: 58–66. 10.1016/j.tvjl.2005.03.008 16772132

[r56] Lardy G , Stoltenow CL , Johnson R , Boyles S , Fisher G , Wohlgemuth K and Lundstrom D 2008 Livestock and water. NDSU Extension Service: North Dakota, USA.

[r57] Larsson A and Müller CE 2018 Owner reported management, feeding and nutrition-related health problems in Arabian horses in Sweden. Livestock Science 215: 30–40. 10.1016/j.livsci.2017.03.001

[r58] Leme DP , Parsekian ABH , Kanaan V and Hötzel MJ 2014 Management, health, and abnormal behaviors of horses: A survey in small equestrian centers in Brazil. Journal of Veterinary Behavior: Clinical Applications and Research 9: 114–118. 10.1016/j.jveb.2014.01.004

[r59] Lesimple C , Fureix C , De Margerie E , Sénèque E , Hervé M and Hausberger M 2012 Towards a postural indicator of back pain in horses (*Equus caballus*). PLoS One 7: e44604. 10.1371/journal.pone.0044604 PMC3436792 22970261

[r60] Leśniak K , Williams J , Kuznik K and Douglas P 2017 Does a 4–6-week shoeing interval promote optimal foot balance in the working equine? Animals 7. 10.3390/ani7040029 PMC5406674 28353665

[r61] McGreevy P 2004 Equine Behaviour. Saunders: London, UK.

[r62] McGreevy PD , Cripps PJ , French NP , Green LE and Nicol CJ 1995 Management factors associated with stereotypic and redirected behaviour in the Thoroughbred horse. *Equine Veterinary Journal* 86–91. 10.1111/j.2042-3306.1995.tb03041.x 7607155

[r63] Mejdell C and Bøe K 2005 Responses to climatic variables of horses housed outdoors under Nordic winter conditions. Canadian Journal of Animal Science 85: 301–308. 10.4141/A04-066

[r64] Mills D and Nankervis K 1999 Equine Behaviour: Principle and Practice. Blackwell Science Ltd: London, UK.

[r65] Morgan K 1998 Thermoneutral zone and critical temperatures of horses. Journal of Thermal Biology 23: 59–61. 10.1016/S0306-4565(97)00047-8

[r66] Muñoz AL , Ainardi CF , Rehhof VC , Cruces LJ , Ortiz RR and Briones LM 2014 Prevalence of stereotypies in thoroughbred racehorses at Club Hipico Concepción, Chile. Revista MVZ Cordoba 19: 4259–4268. 10.21897/rmvz.88

[r67] Murray JAMD , Bloxham C , Kulifay J , Stevenson A and Roberts J 2015 Equine nutrition: A survey of perceptions and practices of horse owners undertaking a massive open online course in equine nutrition. Journal of Equine Veterinary Science 35: 510–517. 10.1016/j.jevs.2015.02.005

[r68] Murray RC , Walters JM , Snart H , Dyson SJ and Parkin TDH 2010 Identification of risk factors for lameness in dressage horses. The Veterinary Journal 184: 27–36. 10.1016/j.tvjl.2009.03.020 19369100

[r69] Nadeau J 2006 Pasture: evaluation and management of existing pasture. Extension Articles 6. https://opencommons.uconn.edu/ansc_ext/6

[r70] Ninomiya S , Kusunose R , Sato S , Terada M and Sugawara K 2004 Effects of feeding methods on eating frustration in stabled horses. Animal Science Journal 75: 465–469. 10.1111/j.1740-0929.2004.00214.x

[r71] Pratt R , Putman R , Ekins J and Edwards P 2016 Use of habitat by free-ranging cattle and ponies in the New Forest, Southern England. Applied Ecology 23: 539–557. 10.2307/2404035

[r72] Ruet A , Lemarchand J , Parias C , Mach N , Moisan MP , Foury A , Briant C and Lansade L 2019 Housing horses in individual boxes is a challenge with regard to welfare. Animals 9: 1–19. 10.3390/ani9090621 PMC6770668 31466327

[r73] Rutberg AT and Greenberg SA 1990 Dominance, aggression frequencies and modes of aggressive competition in feral pony mares. Animal Behaviour 40: 322–331. 10.1016/S0003-3472(05)80927-3

[r74] Sankey C , Richard-Yris M-A , Leroy H , Henry S and Hausberger M 2010 Positive interactions lead to lasting positive memories in horses, *Equus caballus* . Animal Behaviour 79: 869–875. 10.1016/j.anbehav.2009.12.037

[r75] Sarrafchi A and Blokhuis HJ 2013 Equine stereotypic behaviors: Causation, occurrence, and prevention. Journal of Veterinary Behavior: Clinical Applications and Research 8: 386–394. 10.1016/j.jveb.2013.04.068

[r76] Siciliano PD 2012 Estimation of pasture dry matter intake and its practical application in grazing management for horses. In: Zimmermann N (ed) Proceedings of the Tenth Mid-Atlantic Nutrition Conference pp 9–12. Maryland Feed Industry Council, Timonium, MA, USA.

[r77] Sigurjónsdóttir H , van Dierendonck MC , Snorrason S and Thórhallsdóttir AG 2003 Social relationships in a group of horses without a mature stallion. Behaviour 140: 783–804. 10.1163/156853903322370670

[r78] Sigurjónsdóttir H and Haraldsson H 2019 Significance of group composition for the welfare of pastured horses. Animals 9: 14. 10.3390/ani9010014 30621272 PMC6356279

[r79] Snoeks MG , Moons CPH , Ödberg FO , Aviron M and Geers R 2015 Behavior of horses on pasture in relation to weather and shelter: A field study in a temperate climate. Journal of Veterinary Behavior: Clinical Applications and Research 10: 561–568. 10.1016/j.jveb.2015.07.037

[r80] Søndergaard E and Ladewig J 2004 Group housing exerts a positive effect on the behaviour of young horses during training. Applied Animal Behaviour Science 87: 105–118. 10.1016/j.applanim.2003.12.010

[r81] Souris A , Kaczensky P , Julliard R and Walzer C 2007 Time budget, behavioral synchrony and body score development of a newly released Przewalski’s horse group *Equus ferus przewalskii* in the Great Gobi B strictly protected area in SW Mongolia. Applied Animal Behaviour Science 107: 307–321. 10.1016/j.applanim.2006.09.023 22064904 PMC3207227

[r82] Stanley CR , Mettke-Hofmann C , Hager R and Shultz S 2018 Social stability in semi-feral ponies: networks show interannual stability alongside seasonal flexibility. Animal Behaviour 136: 175–184. 10.1016/j.anbehav.2017.04.013

[r83] Thorne JB , Goodwin D , Kennedy MJ , Davidson HPB and Harris P 2005 Foraging enrichment for individually housed horses: Practicality and effects on behaviour. Applied Animal Behaviour Science 94: 149–164. 10.1016/j.applanim.2005.02.002

[r84] Tyler SJ 1972 The behaviour and social organisation of the New Forest Ponies. Animal Behaviour Monographs 5: 87–196.

[r85] van Dierendonck MC , Sigurjónsdóttir H , Colenbrander B and Thorhallsdóttir A 2004 Differences in social behaviour between late pregnant;/post-partum and barren mares in a herd of Icelandic horses. Applied Animal Behaviour Science 89: 283–297. 10.1016/j.applanim.2004.06.010

[r86] van Eps A 2012 Progress towards effective prevention and therapy for laminitis. Equine Veterinary Journal 44: 746–748. 10.1111/j.2042-3306.2012.00667.x 23106627

[r87] Visser E , Neijenhuis F , De Graaf-Roelfsema E , Wesselink H , De Boer J , van Wijhe-Kiezebrink M , Engel B and van Reenen CG 2014 Risk factors associated with health disorders in sport and leisure horses in the Netherlands. Journal of Animal Science 92: 844–855. 10.2527/jas2013-6692 24352963

[r88] Waring G 2003 Horse Behaviour, 2nd Edition. Noyes/William Andrew Publishing: Norwich, UK.

[r89] Wheeler R , Christley R and McGowan C 2002 Prevalence of owner-reported respiratory disease in Pony Club horses. Veterinary Record 150: 79–81. 10.1136/vr.150.3.79 11837591

